# ANTIBIOTIC DISCONTINUATION IN NON-ICU HOSPITALIZED PATIENTS WITH RESPIRATORY TRACT INFECTIONS HAVING LOW PROCALCITONIN VALUES

**DOI:** 10.51894/001c.122896

**Published:** 2024-08-30

**Authors:** Andrew Victor, Kaitlyn Zurek

**Affiliations:** 1 Family Medicine Ascension Genesys

## 37

### INTRODUCTION

As of March 2023, Ascension removed procalcitonin as an orderable lab test. Their rationale was based on limited evidence of antibiotic management practices changing based on the procalcitonin result. However, most of the data acquired was in ICU or septic patients which is not what procalcitonin is validated for. Ascension Genesys has shown longer duration of antibiotic use above the regional and national averages (per internal data review). Procalcitonin is most accurate in patients with respiratory tract infections which is a patient demographic that has not been studied within Ascension Genesys at a high enough volume. Ascension’s decision was based on a random sample of 57 procalcitonin values from a total of 3210 procalcitonin values ordered over 1 year (Oct 2021 – Sept 2022).

### AIMS/OBJECTIVES

To determine the clinical relevance of low procalcitonin values for antibiotic de-escalation. This study will assess whether there still may be a need for procalcitonin as a diagnostic test in a specific population subgroup as well as whether the lab test is being used appropriately.

### METHODS

Retrospective investigation of all procalcitonin values gathered from Ascension Genesys in hospitalized non-ICU patients with respiratory tract infections from February 2022 – February 2023. Statistical analysis was performed of the acquired data and rates of antibiotic discontinuation within 24 hours of the low procalcitonin value.

### RESULTS

A total of 250 procalcitonin values were obtained for the patient population selected. 185/250 were considered low (procalcitonin value <0.25 ng/mL), or 74%. Of the 185 low procalcitonin values, 96.7% were deemed infections likely viral in origin. Finally, of the low procalcitonin values, only 36.2% had antibiotics discontinued within 24 hours of the result.

### DISCUSSIONS/CONCLUSIONS

This study shows that over 1 year of procalcitonin ordering in a population of hospitalized non-ICU patients with respiratory tract infections, only 36.2% of the low procalcitonin orders led to early antibiotic discontinuation. However, the low procalcitonin values did correlate with an overwhelming majority of probable viral infections. This study supports the removal of procalcitonin as an order due to inappropriate use.

